# Reply to: Role of ambient humidity underestimated in research on correlation between radioactive decay rates and space weather

**DOI:** 10.1038/s41598-022-06179-7

**Published:** 2022-02-15

**Authors:** Víctor Milián-Sánchez, Miguel E. Iglesias-Martínez, Felix Scholkmann, Pedro Fernández de Córdoba, Juan C. Castro-Palacio, Sarira Sahu, Antonio Mocholí, Ferrán Mocholí, G. Verdú, Valeriy A. Kolombet, Victor A. Panchelyuga

**Affiliations:** 1 Institute for Industrial, Radiophysical and Environmental Safety, Universitat Politècnica de València, Camino de Vera, s/n, 46022 Valencia, Spain; 2 Grupo de Modelización Interdisciplinar, InterTech, Instituto Universitario de Matemática Pura y Aplicada, Universitat Politècnica de València, Camino de Vera, s/n, 46022 Valencia, Spain; 3 Research Office for Complex Physical and Biological Systems, 8006 Zurich, Switzerland; 4 Department of Electrical Engineering, Electronics, Automation, and Applied Physics, Technical University of Madrid, Ronda de Valencia, 3, 28012 Madrid, Spain; 5 Instituto de Ciencias Nucleares, Universidad Nacional Autónoma de México, Circuito Exterior, C.U., A. Postal 70-543, 04510 Mexico, DF Mexico; 6 Traffic Control Systems Group, ITACA Institute, Universitat Politècnica de València, Camino de Vera, s/n, 46022 Valencia, Spain; 7 Chemical and Nuclear Engineering Department, Universitat Politècnica de València, Camino de Vera, s/n, 46022 Valencia, Spain; 8 Institute of Theoretical and Experimental Biophysics, Russian Academy of Science, Moscow Region, Pushchino, Russia 142290

**Keywords:** Experimental nuclear physics, Nuclear astrophysics

**replying to** : S. Pommé and K. Pelczar; *Scientific Reports* 10.1038/s41598-022-06171-1 (2022).

## Introduction

In their comment entitled “Role of ambient humidity underestimated in research on correlation between radioactive decay rates and space weather”, Pommé and Pelczar^1^ discussed the findings of correlations between measured radioactive decay rates and space weather that we reported recently in this journal^2^. In the following we would like to make some remarks and clarify several important aspects raised.

Regarding the aspects to clarify, first, it all seems the results of our work were interpreted differently from what we really wanted to express. We make some key clarifications as follows:

(i) In reference^1^, the authors interpreted our results as providing indications for a “causal correlation between space weather and radioactive decay”. In fact, we just provided examples where nuclear decay *measurements* (and thus not nuclear decay per se) were found to be correlated with space weather indices.
(ii) Again in Ref.^1^, our research is considered “exploratory” but, in our view, we go beyond and provide a detailed correlation analyses based on a wide range of empirical data. We brought up these results to a broader community as it may be relevant for the various findings reported so far about unexpected variations in nuclear decay data measurements.
(iii) Pommé and Pelczar regard the nuclear decay’s “correlation with space weather as highly speculative”. In this report, we would like to point out once again that what we have found was a correlation between nuclear decay *measurements* and space weather when measured inside the MFC. There may be different reasons for that such as measurements (the electronics) may have been distorted by a still-unknown-to-us factor. We certainly need to do more experiments to clarify this issue.

We agree that in normal circumstances (i.e. outside the cage) there is no correlation between space weather and radioactive decay rates *measurements*. In our first paper^3^, we investigated the impact of room temperature and wet air density on the nuclear decay (see Fig. 2 in Ref.^3^) and cable capacitance measurements (see Figs. 10, 13 in Ref.^3^) and found no obvious correlation. It was then when Scholkmann^4^ discovered that two space weather variables were often correlated with them, which lead to the further analysis presented in Ref.^2,5^. Thus, classical environmental factors were considered first to be the possible reasons for the observed fluctuations and afterwards non-classical ones were explored.

Pommé and Pelczar developed a “toy model”^6^ which shows that the calculated humidity correlates generally well with our measurements of nuclear decay and capacitance.

In order to get deeper insights into the significance of the toy model, we made a systematic analysis of the correlations (see Results in Table 1) considering all the published data in references^2,3^. In Table 1, the first column refers to the figure number in Ref.^3^. The next two columns are the statistical analysis between the decay rates and the actual relative humidity.

**Table 1:** Correlation analyses.

Figures in Ref.^3^	Cpm/RH (actual)		Cpm/Dcx		Cpm/N	
	Pearson correlation coefficient, Bayes factor, *p*-value	Spearman correlation coefficient, Bayes factor, *p*-value	Pearson correlation coefficient, Bayes factor, *p*-value	Spearman correlation coefficient, Bayes factor, *p*-value	Pearson correlation coefficient, Bayes factor, *p*-value	Spearman correlation coefficient, Bayes factor, *p*-value
1c	− 0.02537	0.00696	0.1165	0.0155	− 0.0001	− 0.0324
	0.16778	0.1744	0.1892	0.1671	0.1667	0.1684
	0.9131	0.7555	0.6148	0.9444	0.9995	0.8845
1d	− 0.1434	− 0.0775	0.2441	0.1731	**0.4103**	**0.3527**
	0.1861	0.1175	0.6581	0.2512	**30.59**	**6.0617**
	0.2541	0.5352	0.0500	0.1659	**0.0007**	**0.0048**
3a	**0.4409**	**0.5413**	− **0.3937**	− **0.3391**	**0.3754**	**0.3285**
	**1021.21**	**301,041.63**	**127.91**	**17.021**	**62.15**	**12.045**
	** < 0.0001**	** < 0.0001**	**0.0001**	**0.0014**	**0.0003**	**0.0019**
3b	0.1675	0.2043	0.1117	− 0.0707	0.1598	0.1966
	0.2106	0.2483	0.1749	0.1601	0.2043	0.2393
	0.4134	0.3069	0.5868	0.7234	0.4354	0.3256
4a	0.2653	0.2736	− 0.1863	− **0.4744**	− 0.0309	− 0.0187
	0.6543	0.7369	0.2591	**57.3897**	0.1112	0.1095
	0.0573	0.0507	0.1859	**0.0007**	0.8278	0.8935
4b	− **0.4774**	− **0.4569**	**0.3811**	**0.4132**	− 0.1137	− 0.1290
	**30.7619**	**18.1159**	**3.4206**	**6.5766**	0.1526	0.1656
	**0.0008**	**0.0022**	**0.0090**	**0.0056**	0.4517	0.3865
4c	0.1880	0.2063	− 0.0814	0.1640	− 0.2872	− 0.3232
	0.2308	0.2492	0.17116	0.2112	0.3847	0.4926
	0.3902	0.3330	0.7117	0.4416	0.1839	0.1294
4d	**0.2852**	**0.2741**	− **0.2742**	− **0.3306**	− 0.0898	− 0.0754
	**17.164**	**11.058**	**11.094**	**128.212**	0.1163	0.0995
	**0.0009**	**0.0016**	**0.0014**	**0.0001**	0.3039	0.3863
5	**0.5032**	**0.5251**	− **0.5344**	− **0.4259**	0.2966	0.1235
	**5.1654**	**7.489**	**8.8337**	**1.6935**	0.4554	0.1789
	**0.0074**	**0.0074**	**0.0041**	**0.0299**	0.1329	0.5288
6a	0.1479	0.1817	0.1095	0.1007	**0.5139**	**0.4871**
	0.1931	0.2232	0.1700	0.1660	**7.0613**	**4.4903**
	0.4524	0.3451	0.5789	0.6008	**0.0051**	**0.0114**
6b	0.0722	− 0.1867	0.0197	− 0.1630	0.1781	0.1993
	0.1605	0.2287	0.1517	0.2069	0.2201	0.2425
	0.7258	0,3503	0.9236	0,4149	0.3839	0.3190
6c	-0.0412	0.0142	− 0.0847	− 0.1106	− **0.2497**	− **0.2275**
	0.0741	0.0665	0.1099	0.1584	**6.5778**	**2.9416**
	0.6208	0.8633	0.3089	0.1826	**0.0024**	**0.0061**
6d	− 0.1624	− 0.1407	0.0403	0.0567	**0.3276**	**0.4011**
	0.2295	0.1836	0.0999	0.1053	**4.0538**	**31.325**
	0.1822	0.2456	0.7419	0.6400	**0.0060**	**0.0009**
10	− **0.3534**	− **0.3841**	**0.4138**	**0.4789**	0.0704	**0.2185**
	**58,552.25**	**889,346.499**	**16,701,321.0**	**32.36E7**	0.0917	**8.8725**
	** < 0.0001**	** < 0.0001**	** < 0.0001**	** < 0.0001**	0.3082	**0.0015**

The first column refers to the figure number in Ref.^3^. The next two columns are the statistical analysis results between the decay rates and the actual relative humidity, and the remaining four columns include the correlation analysis results with respect to geomagnetic activity (GMA) and cosmic-ray activity (CRA).

Values marked in bold refer to significant correlations or anticorrelations; *p*-value cut-off is 0.05.

For every pair of data, the following was calculated: (a) the Pearson correlation coefficient, (b) the Spearman correlation coefficient, (c) a Bayes test to consider true or false the hypothesis regarding the correlation values obtained, and (d) a test of statistical significance to consider relevant or non-relevant correlation values (*p*-value)^7^. The values in bold inside the highlighted boxes are the cases in which there are significant correlations (or anticorrelations).

It can be noticed that the possible hypothesis about the correlation of the measured data with the relative humidity (RH) is not always true (*p* < 0.05; *B* > 1); therefore, it cannot be overestimated and concluded that the RH is the independent variable that completely describes the process. Results in Ref.^1^ confirm that the RH is a variable that certainly plays a role in the observed correlations but not the only one as shown by our results in Table 1. What really happens could be rationalized by looking into the interplay between RH, geomagnetic activity (GMA) and cosmic-ray activity (CRA)^3,8^.

As pointed out above, in a new experiment (performed during the months of July and September 2020) we checked again how capacitance changes inside an MFC are related to variations in GMA and CRA^9^.
